# Toward a consensus on dyslexia: findings from a Delphi study

**DOI:** 10.1111/jcpp.14123

**Published:** 2025-02-25

**Authors:** Julia M. Carroll, Caroline Holden, Philip Kirby, Paul A. Thompson, Margaret J. Snowling, the Dyslexia Delphi Panel, Shenaz Bagshaw, Gill Brackenbury, Sophie Brigstocke, Lia Castiglione, Batul Daulby, Helen Duncan, Lynne G. Duncan, Gad Elbeheri, Julian Elliott, James Gilchrist, Debbie Gooch, Janet Goring, Sara Graham, Jennie Guise, Claire Harvey, Marianna E. Hayiou‐Thomas, Elisabeth Herbert, Charles Hulme, Brian Lamb, Jeremy Law, Michelle Luciano, Catherine Manning, Nancy Mather, Genevieve McArthur, Cammie McBride, Anne McLoughlin, Sharon McMurray, Jessica Milligan, Kristina Moll, Sally‐Ann Morrison, Brahm Norwich, Hetal Patel, Chivonne Preston, Franck Ramus, Gavin Reid, Sarah Sainty, Rob Savage, Rachel Simpson, Paul Strange, Caro Strover, Jill Swinhoe, Joel Talcott, Rebecca Tall, Jenny M. Thomson, Elsje van Bergen, Alice Voute, Richard K. Wagner, Jane Warren, Bonnie Wing‐Yin Chow, Tanya Zybutz, John Rack

**Affiliations:** ^1^ School of Education University of Birmingham Birmingham UK; ^2^ SpLD Assessment Standards Committee (SASC); ^3^ School of Education, Communication and Society Kings College London London UK; ^4^ School of Social Policy and Society University of Birmingham Birmingham UK; ^5^ Department of Experimental Psychology University of Oxford Oxford UK

**Keywords:** Dyslexia, neurodevelopmental disorders, reading disorder, specific learning difficulties, spelling disorder

## Abstract

**Background:**

Dyslexia is one of the most common neurodevelopmental disorders. There have been many definitions over the past century, and debate continues as to how dyslexia should be defined. This debate contributes to confusion and misinformation. We move beyond the debate by establishing areas of consensus among a wide range of experts.

**Methods:**

We conducted a Delphi study with a panel of dyslexia experts, including academics, specialist teachers, educational psychologists, and individuals with dyslexia, asking them for their views on a set of key statements about dyslexia. We carried out two survey rounds, in each case accepting statements with greater than 80% consensus and reviewing and revising other statements using feedback from the expert panel. This was followed by discussion with a subset of the panel around a few statements with marginal consensus.

**Results:**

Forty‐two statements were ultimately accepted. In the current paper we review those statements that pertain to a definition of dyslexia, demonstrate how they align with the research literature, and build on previous definitions of dyslexia.

**Conclusions:**

There was considerable consensus in our expert panel that dyslexia is a difficulty in reading and spelling, associated with multiple factors, and that it frequently co‐occurs with other developmental disorders. It was agreed that difficulties in reading fluency and spelling are key markers of dyslexia across different ages and languages. We conclude with a proposed new definition of dyslexia.

## Contemporary concepts of dyslexia: A Delphi study

Definitions of neurodevelopmental disorders allow consistent identification and support of those affected. A successful definition should reflect current scientific knowledge of the condition and be translatable into clear and workable guidelines for assessment, identification, and education policy. For these reasons, definitions of learning difficulties, such as ‘dyslexia,’ are regularly revised and updated. This paper reports findings from a Delphi project which obtained a consensus definition of dyslexia from a multidisciplinary group of professionals. Throughout we use ‘disorder’ to align with contemporary terminology in diagnostic manuals, and of other common conditions with which dyslexia co‐occurs, such as attention deficit hyperactivity disorder (ADHD) and developmental language disorder (DLD), though the term is not without controversy, and some jurisdictions use alternatives (SASC, 2022).

The term ‘dyslexia’ was coined by the German ophthalmologist Rudolf Berlin in the 1880s (see Kirby & Snowling, 2022). Since then, several definitions have vied for prominence, including “congenital word blindness” (Kussmaul, 1877; Pringle Morgan, 1896). James Hinshelwood (1896), another ophthalmologist, called dyslexia an “inability to interpret written and printed language.” Hinshelwood was confident that dyslexia existed independent of other abilities, a position codified in 1968 by the World Federation of Neurology:

a disorder in children who, despite conventional classroom experience fail to attain language skills of reading, writing, and spelling commensurate with their intellectual abilities.

It was not until the 1980s that researchers indicated dissatisfaction with this discrepancy definition (Dyck et al., 2004). Later, Stanovich and Siegel (1994) argued that the underlying causes of literacy difficulties did not vary between individuals whose intellectual abilities were discrepant vs. non‐discrepant; rather, they were associated with phonological difficulties irrespective of IQ.

Following this, the International Dyslexia Association definition (IDA, 2002; Lyon, Shaywitz, & Shaywitz, 2003) removed reference to the discrepancy approach, instead introducing the notion of unexpectedness in the context of cognitive abilities and education:

Dyslexia is a specific learning disability that is neurobiological in origin. It is characterized by difficulties with accurate and/or fluent word recognition and by poor spelling and decoding abilities. These difficulties typically result from a deficit in the phonological component of language that is often unexpected in relation to other cognitive abilities and the provision of effective classroom instruction. Secondary consequences may include problems in reading comprehension and reduced reading experience that can impede the growth of vocabulary and background knowledge.

While retaining the emphasis on ‘specificity,’ some classification systems avoid the term dyslexia. ICD 11 (World Health Organization, 2018) uses “developmental learning disorder with impairment in reading”, and DSM‐5 (American Psychiatric Association, 2013) uses “specific learning disorder in reading.” The two definitions differ regarding the role of discrepancy relative to intellectual abilities: ICD‐11 requires that reading is below expectations for age *and* intellectual ability, while DSM‐5 moves away from the diagnostic discrepancy between intellectual ability and literacy attainment, focusing instead on ‘unexpected’ levels of attainment. Neither definition addresses causes.

The emphasis in these definitions on specificity is at odds with recent research, which highlights the extent to which dyslexia co‐occurs with other disorders (Carroll, Maughan, Goodman, & Meltzer, 2005; Landerl & Moll, 2010). In addition, the focus on reading attainment does not consider the changing demands on literacy skills at different developmental stages or the potential role of intervention and support. A definition based only on attainment implies that if an individual receives intervention and improves, the diagnosis may no longer apply. However, clinical experience and parent reports suggest that if these individuals have additional support removed, they will often fall behind their peers again (Thompson, 2021).

A definition widely used in the UK education system is Rose (2009):

Dyslexia is a learning difficulty that primarily affects the skills involved in accurate and fluent word reading and spelling. Characteristic features of dyslexia are difficulties in phonological awareness, verbal memory, and verbal processing speed. Dyslexia occurs across the range of intellectual abilities. It is best thought of as a continuum, not a distinct category, and there are no clear‐cut‐off points. Co‐occurring difficulties may be seen in aspects of language, motor coordination, mental calculation, concentration, and personal organization, but these are not, by themselves, markers of dyslexia. A good indication of the severity and persistence of dyslexic difficulties can be gained by examining how the individual responds or has responded to well‐founded intervention.

This definition aligns with the approach of DSM‐5, highlighting that dyslexia “occurs across the range of intellectual abilities” and acknowledging that it lies on a continuum; it also notes poor response to intervention as an indicator. In taking account of individual differences between people, the definition also references co‐occurring difficulties.

Although Rose drew on empirical evidence, Elliott and Grigorenko (2014) argue that there is no meaningful way of identifying a dyslexic subgroup within the larger pool of those who struggle with decoding text. Elliott (2020) went further to argue there are no ‘specialized, morally or ethically defensible interventions’ that are differentially appropriate for these two groups. Hence, he argues that there is no value in diagnostic assessment of dyslexia: individuals should be supported according to their literacy difficulties, rather than trying to label a subgroup of poor readers. Notably, Elliott (2020) focuses on reading and decoding difficulties, rather than spelling difficulties, which are often persistent in dyslexia (Maughan et al., 2009).

A negative consequence of this so‐called ‘dyslexia debate’ is that policymakers do not receive a clear message concerning the educational needs of individuals with persistent literacy difficulties. More generally, practitioners are confronted with changing conceptualizations of ‘dyslexia’ as new research evidence accumulates (Snowling, Nation & Hulme, 2020). One recent such proposal is that dyslexia is better explained by a constellation of strengths and weaknesses rather than “core deficits” (e.g., Catts et al., 2024). According to this view, while phonological difficulties remain highly relevant, dyslexia has a multifactorial basis, and other factors, such as limitations in oral language and processing speed, and environmental factors such as poverty and trauma, may play an important role.

Changing conceptualizations in the research literature are not necessarily aligned with the views and experiences of practitioners, and the current study aims to find consensus across both groups as well as include the opinions of those with personal experience of dyslexia. Clearly the definition of dyslexia has implications not only for who gets ‘diagnosed’ but also for prevalence estimates; reported rates vary between about 5%–20%, but these depend upon the cut‐off criteria used to define reading disability. Thus, Wagner and colleagues (e.g., Wagner et al., 2020) have argued that prevalence is better described as a distribution depending on severity rather than as a single value and have shown that ‘unexpectedly’ poor readers can be found at all levels of reading dis/ability. Moreover, the stability of the defining characteristics that lead to identification is important, and evidence shows that this is better when several measures are used as markers of dyslexia, rather than a single measure of reading (Spencer et al., 2014).

In the UK there have been two important consultation exercises on the identification of dyslexia in children and adults that formed a backdrop to this Delphi study. The first of these, initiated by the UK's Specific Learning Difficulties (SpLD) Assessment Standards Committee (SASC), focused on good practice in dyslexia assessment, involving a wide range of respondents from different backgrounds. In parallel in 2021, the British Psychological Society (BPS) produced SpLD assessment guidance for psychologists. Key questions for both groups were (i) How should dyslexia be described and defined? (ii) What criteria should be used to identify dyslexia? (iii) What types of assessment and which interventions best support children and adults with literacy (and related) difficulties, including dyslexia?

The present Delphi study allows us to test key ideas from these consultations and from recent research to propose an agreed definition of and identification criteria for dyslexia. The Delphi process involves a set of iterative ‘rounds’ in which an expert panel are asked to rate and give their opinions on statements relating to a specific topic (Barrett & Heale, 2020). The aim is to build consensus, and hence, all responses are anonymous. The current Delphi study was inspired by a similar project for DLD (CATALISE; Bishop et al., 2016; Bishop et al., 2017) and addresses five broad areas: the nature of dyslexia, experiences of dyslexia, why and when to assess, what to assess, and identification criteria. This paper reports particularly on the first two areas.

## Method

### Participants

Dyslexia has been the subject of substantial interdisciplinary research, so it was important to ensure the panel represented a broad range of expertise. Moreover, given recent debates regarding dyslexia's nature and causes and best practice in assessment, it was important to include in the moderating group individuals from the ongoing working groups (JC and CH). Invitations were issued to representatives working in education, psychology, and occupational support, and to individuals with lived experience of dyslexia. Individuals were drawn from the four UK nations (England, Scotland, Wales, and Northern Ireland; *N* = 32) and from a wide variety of other countries where dyslexia has been studied recently (Australia, Canada, Egypt, Finland, France, Germany, Hong Kong, Kuwait, Netherlands, Norway, Portugal, Sweden, USA). Seventy‐one participants accepted and formed the expert panel. Of these, 58 provided responses to the survey in Round 1 and 57 in Round 2. Our panel was predominantly female (*n* = 41), with 17 males. The breakdown of panel members by country and discipline is shown in Table 1.

**Table 1:** Demographic details of the panelists in the Delphi study

Country	Profession	Stakeholder groups represented
England (73%) Scotland (9%) Wales (2%) Northern Ireland (2%) USA (2%) Europe (7%) Other (Egypt, Finland, France, Germany, Hong Kong, Kuwait, Netherlands, Norway, Portugal, Sweden) (5%)	44% Academic 9% Educational Psychologist 27% specialist teacher and/or assessor 20% other, eg., adults with dyslexia; parents of children with dyslexia; CEOs of dyslexia organizations.	PATOSS (Professional Association of Teachers of Students with Specific Learning Difficulties) SASC (SpLD Assessment Standards Committee) British Dyslexia Association Dyslexia Action Working with Dyslexia Helen Arkell Dysguise

### Materials

Fifty‐five statements were assembled from three sources: *The Science of Reading* (Snowling, Hulme & Nation, 2022), *The Dyslexia Debate* (Elliott & Grigorenko, 2014), and responses to a working group and subsequent consultation paper (SASC, 2022). The moderating group (JC, CH, PK, MS) met three times to decide on included statements. These statements were modified in response to two sets of feedback from the expert panel.

### Procedure

Ethical Clearance for the study was obtained from the Coventry University Ethics Committee. All participants gave informed consent for their participation in the survey.

**Figure 1:**
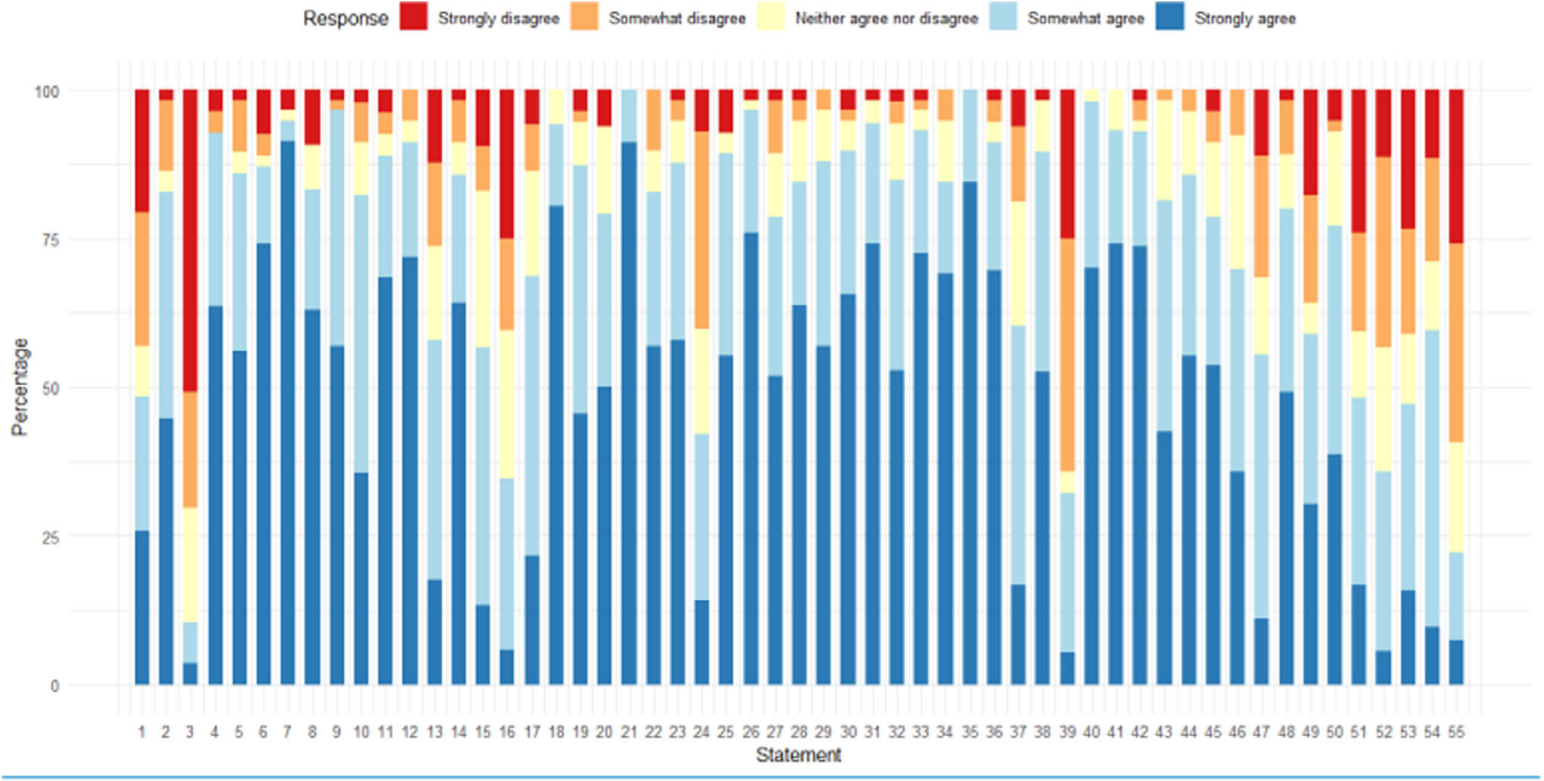
Stacked bar chart showing percentages of responses in each category across all statements in Round 1

**Figure 2:**
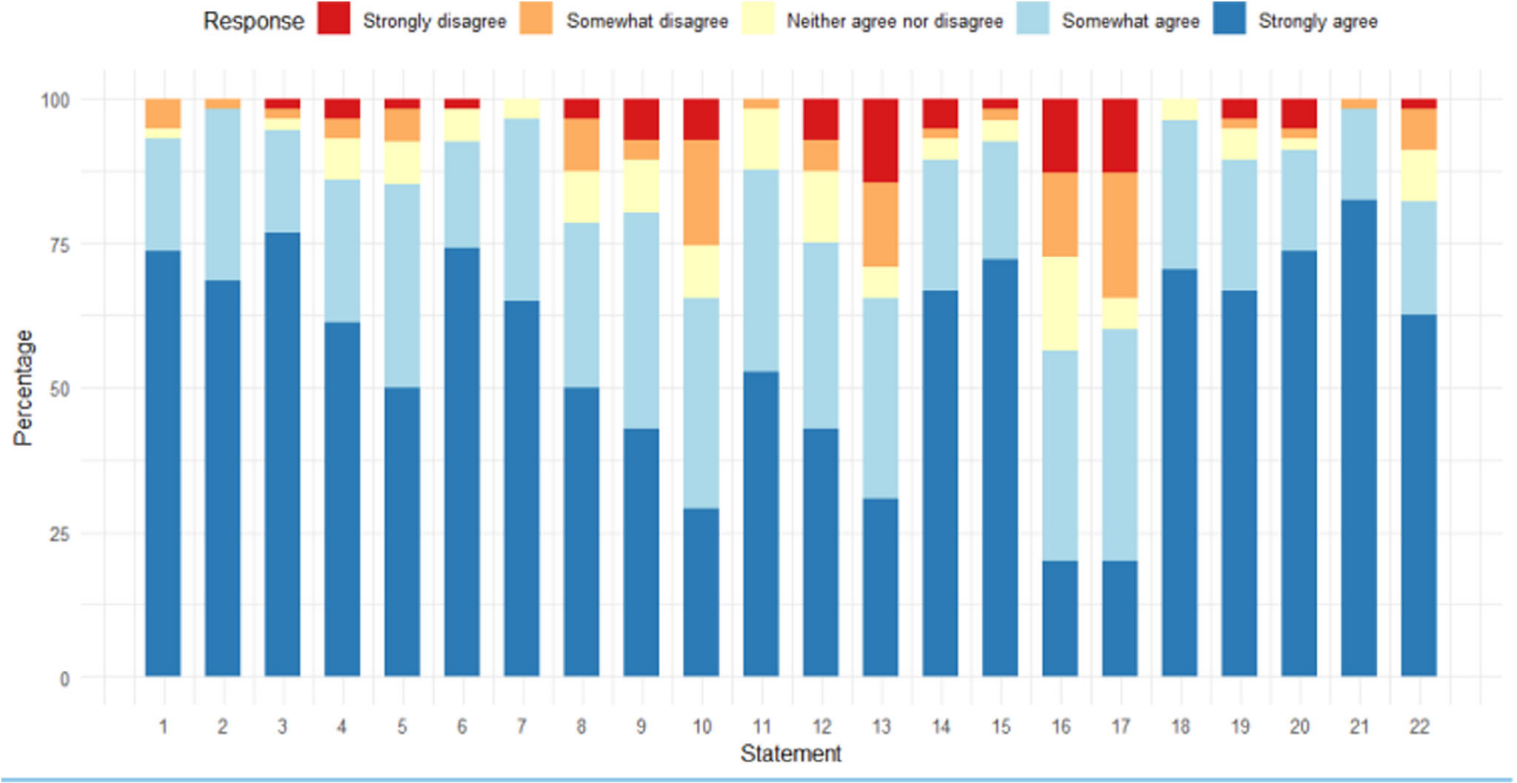
Stacked bar chart showing percentages of responses in each category across all statements in Round 2

Round 1 of the survey was issued in May 2023 using the Qualtrics platform with 4 weeks for responses. The survey began with an introduction to the study and demographic questions. Panel members were then asked to provide their views on the statements, rating each one on a five‐point Likert scale ‐“Strongly Disagree” to “Strongly Agree”‐ or alternatively, could select “No opinion/do not know”. Space was provided after each statement for further comments. Figure 1 shows levels of agreement for each statement in Round 1, while Figure 2 shows levels of agreement for each statement in Round 2. Data giving agreement levels for each statement are provided on the Open Science Framework (OSF: https://osf.io/ey8rs/).

Data were analyzed and reports generated using R statistical software (4.2.3–2023‐03‐15 ucrt; R Core Team, 2023). Anonymized data, reports, and R scripts are on the OSF (https://osf.io/vhxgf/).

After data collection, the independent data controller (PT) collated responses for each statement into an anonymized report for the moderators and into individual reports for each panel member. Individual reports contained the expert's and panel's quantitative responses and all (anonymized) panel comments. Hence, the panel could reflect on their position with reference to the other panel members' responses in Round 2. Meanwhile, using the responses and comments, the moderators reworded, amalgamated, or removed statements as necessary.

Details of all the modifications and decisions are published on the OSF. The threshold for consensus was set at overall agreement ratings (strongly agree, somewhat agree) of 80%. In some cases, respondents agreed with the statement but suggested minor rewording or clarification, which was deemed appropriate, and the statement was accepted with minor modifications.

For statements that did not achieve consensus, the moderators considered the comments to guide whether rewording and retesting or removal of the statement was required. Three statements marginally failed to reach consensus after Round 2 and were refined in a post‐survey meeting with a subset of the panel, along with the overall proposed definition.

## Results and discussion

In this section, we present the final statements after moderation and discussion, including reference to the views of the panel as appropriate.

### Definition of dyslexia

First, we introduce and discuss the agreed statements relating directly to how dyslexia should be defined:

**S16**. In dyslexia, some or all aspects of literacy attainment are weak in relation to age, standard teaching and instruction and level of other attainments.

This statement acknowledges that literacy attainment below expectations is central to dyslexia. Expectations may be based on age, exposure to teaching, quality of instruction or levels of attainments in other areas (unexpectedness). This statement mirrors those found in all four definitions discussed above, though the ways in which ‘weak literacy attainment’ is defined may differ between these (see below).

**S8**. Dyslexia is primarily a set of processing difficulties that affect the acquisition of reading and spelling, despite the educational opportunity to learn these skills.

This statement highlights the role of processing difficulties that underpin the problem in acquiring literacy, rather than the behavioral or surface manifestation of poor reading and spelling. This aligns with IDA and Rose definitions but differs from ICD‐11 and DSM‐5 in using the term ‘dyslexia’.

**S19**. Dyslexic difficulties exist on a continuum and can be experienced to various degrees of severity.

This statement echoes part of the Rose definition, which states that dyslexia is “a continuum, not a distinct category.” There is considerable research evidence to support this assertion (e.g., Snowling, Gallagher, & Frith, 2003). It is not an element highlighted in the IDA, ICD‐11, or DSM‐5 definitions but is alluded to in Catts et al.'s (2024) suggestions for changes to the IDA definition.

**S3**. There are differences in the manifestations of dyslexia, depending on how a language is written (orthography), its sound structure (phonology), grammar, and morphology.

**S10**. Persistent and sometimes severe difficulties in word and non‐word decoding (reading accuracy) are typically observed in children with dyslexia learning to read and spell in English.

**S4**. Across all languages, difficulties in reading fluency and spelling are key markers of dyslexia.

**S11**. While some older children and adults with dyslexia continue to experience word‐level reading problems, others mainly have difficulties in reading and writing fluency and in spelling.

These statements retain the ideas of ‘unexpectedness,’ persistence, and fluency observed in other definitions and move away from both Anglocentric (Share, 2008) and child‐centered perspectives; in non‐English languages, and in older children and adults, basic decoding skills may be adequate while fluency remains an issue. Both the IDA and Rose definitions mention fluency as an important element of dyslexia, though neither explicitly consider it a key marker. However, importantly, prioritizing the importance of reading (and spelling) fluency implies that dyslexia can be present even when aspects of reading and spelling attainments are in the average range. This differs from the ICD and DSM definitions, but is increasingly accepted, particularly when assessing adults and those who have had extensive support to develop their literacy skills. For example, several studies of high‐achieving adults confirm a dyslexic processing profile with reading and spelling difficulties associated with phonological processing weaknesses (Bradshaw, Woodhead, Thompson, & Bishop, 2021; Callens, Tops, & Brysbaert, 2012; Ramus et al., 2003). Moreover, a lack of reading fluency (where fluency is defined as accurate and automatic reading: Hudson, Lane, Pullen, & Torgesen, 2009) appears to be a reliable marker of dyslexia across languages (Pugh & Verhoeven, 2018).

### Intellectual abilities and dyslexia

A topic strongly debated by our Delphi panel was the role of intellectual abilities in identifying dyslexia, reflecting the issues raised in the introduction. Panel members agreed on one issue, however: support should be given to all individuals with literacy difficulties, regardless of definition or accompanying factors.

**S22**. All individuals struggling with literacy require appropriate, targeted intervention, monitoring, and resources.

Two further statements achieved borderline levels of consensus and were discussed further at the sub‐panel meeting.

**S40**. When an individual has general learning difficulties (intellectual disability), applying a dyslexia label may result in too narrow an approach to intervention.

**S41**. Discrepancy between intellectual ability and literacy attainment is a useful indicator of a specific learning difficulty but is not sufficient for a diagnosis in and of itself.

These statements indicate the utility of assessing intellectual abilities when diagnosing and drawing up an intervention plan (see our companion paper; Holden et al., 2025). However, while it was agreed that typically aspects of literacy are weak relative to other educational expectations, the panel does not advocate using intellectual ability as an absolute measure to determine diagnosis or access to services. This is consistent with a multi‐factorial view of dyslexia (see below).

### The etiology of dyslexia

Together the statements relating to etiology caution against assuming that dyslexia is caused by a single factor:

**S1**. A history of dyslexia in the family is a significant risk factor for dyslexia; however, the causes of dyslexia include multiple genetic and environmental factors.

**S5**. Cognitive processes that influence the skills required for literacy are likely to be impaired in dyslexia.

**S2**. Accounts of dyslexia that attribute it to a single cause, such as weak phonology or problems in working memory, do not account for individual variability or the highly overlapping nature of dyslexia with other disorders of learning.

Determining causes of dyslexia (neurobiological or otherwise) is a question for research rather than definition, and therefore ICD‐11 and DSM‐5 do not address them. In contrast, IDA states that dyslexia is ‘neurobiological in origin’ and ‘typically results from a deficit in the phonological component of language’ (see Yeatman, 2022). Convergent evidence indicates that multiple factors have significant independent roles in predicting outcomes in dyslexia (Carroll, Mundy, & Cunningham, 2014; Carroll, Solity, & Shapiro, 2016; Thompson et al., 2015). Catts et al. (2024) call for a broadening of the IDA definition to include multiple causes, in line with the influential multiple deficit view of dyslexia (McGrath, Peterson, & Pennington, 2020; Pennington, 2006). Similarly, here we see strong panel support for the view that multiple causes are involved in dyslexia.

**S7**. The most commonly observed cognitive issue in dyslexia is a difficulty in phonological processing (i.e., in phonological awareness, phonological processing speed, or phonological memory). However, phonological difficulties do not fully explain the variability that is observed.

**S6**. Orthographic processing refers to the ability to form and retrieve letters, letter sequences, and spelling patterns, and is commonly impaired in dyslexia.

Despite evidence that there are multiple causes of dyslexia, the quality and weight of evidence indicating that phonological skills, specifically, are causally related to dyslexia (Melby‐Lervåg, Lyster, & Hulme, 2012) is stronger than the evidence for other factors, such as impairments in visual attention, visual processing, or orthographic skills (Ramus et al., 2003); arguably this clarifies the reference to cognitive processes in S5. Phonological awareness here refers to the ability to reflect on and manipulate the sound structure of words, and phonological memory refers to short‐term memory for verbal information, typically measured either by repeating nonwords or lists of words or numbers. Both skills are important markers of dyslexia, though phonological memory has a weaker association with dyslexia than phonological awareness (Melby‐Lervåg et al., 2012). A third processing measure, rapid automatized naming (RAN) of familiar symbols, pictures, or colors is also a key marker of dyslexia in alphabetic and non‐alphabetic writing systems (Araújo & Faísca, 2019). RAN is often referred to as a measure of phonological processing speed (e.g., Wagner, Torgesen, Laughon, Simmons, & Rashotte, 1993), though there remains some debate as to whether this characterization provides a complete explanation of the mechanisms that link RAN to literacy development and dyslexia (Poulsen, Juul, & Elbro, 2015). Some research highlights that RAN involves the serial retrieval of lexical codes in response to visual information, just as reading does (Protopapas, Altani, & Georgiou, 2013). Nonetheless, RAN clearly involves a significant phonological processing component (Lervåg & Hulme, 2009).

Some respondents requested the inclusion of orthographic processing as an additional potential cause of dyslexic difficulties alongside phonological processing. However, while there is good evidence that orthographic processing is impaired in dyslexia (Georgiou, Martinez, Vieira, & Guo, 2021), it is less clear that it is a cause of dyslexia separable from prior literacy experience or phonological processing (Burt, 2006; Deacon, Benere, & Castles, 2012). We therefore suggest orthographic processing difficulties are best regarded as a useful marker for dyslexia and that further research investigates the role of orthographic processing.

Notwithstanding this, although the skills involved in learning to read are similar across alphabetic languages (Caravolas, Lervåg, Defior, Seidlová Málková, & Hulme, 2013), evidence suggests that learning to read in non‐alphabetic systems requires a broader range of skills (Nag, 2022). Moreover, there is a reported dissociation between Hong Kong children's reading of Chinese and English relating to the different demands of the two scripts (McBride, Pan, & Mohseni, 2022).

### Co‐occurrence with other disorders

Recent years have seen burgeoning interest in the frequent co‐occurrence of different disorders of learning (Moll, Snowling, & Hulme, 2020). Neither the IDA, DSM‐5, nor ICD‐11 mention the high levels of co‐occurring difficulties associated with dyslexia as part of the definition, though Rose (2009) does. A neutral statement on ‘comorbidities’ was strongly supported by the Delphi panel:

**S18**. Dyslexia frequently co‐occurs with one or more other developmental difficulties, including developmental language disorder, ADHD, developmental coordination disorder, and dyscalculia.

While co‐occurring difficulties are acknowledged, we note that further research is required to better understand the shared and specific risk factors that account for this co‐occurrence.

### The changing impact of dyslexia over the lifespan

Evidence suggests that dyslexia is persistent through adolescence (Shaywitz et al., 1999) and predicts future literacy through adulthood (Ferrer, Shaywitz, Holahan, & Shaywitz, 2023; Maughan et al., 2009). However, the statements reflected views on its changing impact:

**S14**. Multiple factors influence the impact and trajectory of dyslexia. The manifestations of dyslexia can change over time depending on context.

**S31**. Working memory, processing speed, and orthographic skills can contribute to the impact of dyslexia.

**S20**. After intervention and appropriate support, reading and the associated difficulties of individuals with dyslexia may no longer be experienced as disabling, although they may remain challenging.

**S15**. Protective factors in dyslexia include early and sustained intervention and good oral language, verbal, and nonverbal skills.

**S10. (second sentence)**. Secondary consequences of dyslexia may include problems in reading comprehension and reduced reading experience that can impede growth of vocabulary and background knowledge.

**S17**. Dyslexia can affect the acquisition of other skills, such as mathematics or learning another language.

These statements highlight the dynamic nature of dyslexia (Torppa, Eklund, van Bergen, & Lyytinen, 2015) and the need to take a developmental perspective when considering its impact. Multiple longitudinal studies indicate that there are different patterns of deficits depending on age (Lyytinen et al., 2005; Scarborough, 1990; Snowling, Muter, & Carroll, 2007). There is also variability in presentation depending on the presence of factors such as working memory and (general) processing speed, though there is no evidence that these are causal. Also highlighted were protective factors that reduce the most severe manifestations of reading and spelling difficulties. For example, some children with dyslexia experience significant reading comprehension impairments, whereas others can use well‐developed language skills to avoid such difficulties (e.g., Nation & Snowling, 1998; Snowling, Hulme, & Nation, 2020).

In summary, participants largely agreed about the secondary consequences of dyslexia and that persistent difficulties in reading could affect the acquisition of other skills, such as mathematics (e.g., Snowling, Moll, & Hulme, 2021). The view that the ability to learn a foreign language could be affected in dyslexia is less well‐evidenced. While issues with this are commonly reported (Kormos & Smith, 2023), a recent meta‐analysis of studies examining learning English as a foreign language suggests that findings are highly variable (von Hagen, Kohnen, & Stadie, 2021).

### Common misconceptions

Some of the Delphi statements addressed misconceptions associated with dyslexia. While these do not form a defining characteristic, consensus was considered important to reduce the probability of misinformation.

**S9**. The term developmental dyslexia distinguishes dyslexia with a childhood onset from cases of acquired dyslexia with a neurological cause (such as brain injury).

This statement clarifies the distinction between developmental and acquired dyslexia (see also ICD‐11). Developmental dyslexia is relatively common and generally addressed through educational support and assistive technology, while acquired dyslexia is relatively rare and normally has a clear organic cause. There are important differences in the nature and specificity of the two disorders (Ellis, 1985).

**S12**. While there is suggestive evidence of an association between non‐right‐handedness (left or mixed‐handedness) and dyslexia, the information is not useful for identifying dyslexia.

A link between dyslexia and laterality was proposed almost a century ago by Orton (1925). A recent meta‐analysis (Packheiser et al., 2023) shows that individuals with dyslexia are more likely to be non‐right‐handed, but the size of the effect is relatively small and therefore does not provide diagnostic evidence.

**S13**. Visual stress is a condition in which the visual system appears to be hypersensitive to high‐contrast regular patterns, including lines of black text against a white background. Visual stress is a separate condition from dyslexia, but it can make it difficult to process text and hence may exacerbate reading difficulties.

Dyslexia is sometimes assumed by laypeople to be a visual issue, but research indicates that difficulties with visual stress are separable from dyslexia and should be treated separately (Griffiths, Taylor, Henderson, & Barrett, 2016; Kriss & Evans, 2005).

**S21**. People with dyslexia may develop other skills as an adaptive process to compensate for literacy‐based difficulties. However, there is little evidence to support the idea that dyslexia confers advantages in, for example, creative or visual–spatial skills.

Some prominent individuals with dyslexia are very successful, and some attribute their success to ‘dyslexic thinking’ relating to creativity or visual–spatial thinking (Rooke, 2017). Recent meta‐analyses have not, however, shown a relationship between dyslexia and creativity to hold consistently at a group level (Erbeli, Peng, & Rice, 2022; Majeed, Hartanto, & Tan, 2021). Similarly, a recent meta‐analysis indicated a relative weakness in visuospatial processing for dyslexic groups (Chamberlain, Brunswick, Siev, & McManus, 2018).

#### Formulation of a definition of dyslexia

The statements above give scope to propose an updated definition of dyslexia based on consensus across research and practice. A definition of a learning disorder such as dyslexia should allow researchers and practitioners to establish consistently what should or should not be considered ‘dyslexia,’ what the boundaries to diagnosis should include, and what elements are important in assessment. Thus, it should highlight the key elements of the disorder and reflect the best quality research evidence alongside practitioner experience. Based on the consensus statements, we propose a definition of dyslexia as shown in Table 2.

**Table 2:** Delphi definition of dyslexia

Statement number	Statement
S8	Dyslexia is a set of processing difficulties that affect the acquisition of reading and spelling.
S16	In dyslexia, some or all aspects of literacy attainment are weak in relation to age, standard teaching and instruction, and level of other attainments.
S4	Across languages and age groups, difficulties in reading fluency and spelling are a key marker of dyslexia.
S19	Dyslexic difficulties exist on a continuum and can be experienced to various degrees of severity.
S14	The nature and developmental trajectory of dyslexia depend on multiple genetic and environmental influences.
S17	Dyslexia can affect the acquisition of other skills, such as mathematics, reading comprehension, or learning another language.
S7	The most commonly observed cognitive impairment in dyslexia is a difficulty in phonological processing (i.e. in phonological awareness, phonological processing speed or phonological memory). However, phonological difficulties do not fully explain the variability that is observed.
S31	Working memory, processing speed, and orthographic skills can contribute to the impact of dyslexia.
S18	Dyslexia frequently co‐occurs with one or more other developmental difficulties, including developmental language disorder, dyscalculia, ADHD, and developmental coordination disorder.

## General discussion

The present Delphi study, which surveyed the views of 58 dyslexia experts, allows us to propose a new definition for dyslexia, some elements of which overlap with previous definitions. The Delphi definition retains the idea of difficulties with reading and spelling relative to age, ability, or educational expectations. However, it is less focused on English speakers and children. In line with evidence, we highlight that phonological processing has a causal link to dyslexia, but that other factors also play an important role in explaining variability in presentation. Finally, we note the high rates of co‐occurrence between dyslexia and other developmental difficulties, not only in terms of categorical diagnoses but also with subclinical features of disorders (Carroll et al., 2005; Snowling et al., 2019).

Some critics might argue that a definition that acknowledges multiple causal factors and co‐occurring difficulties creates complexity. We argue that complexity is actually a strength. For theory, it directs research attention toward the most common predictors, or risk factors, for persistently poor reading and spelling. Here there was strong consensus that phonological difficulties are predictive of difficulties in word reading and spelling, and while the expression of these difficulties varies in different orthographies, a persistent and potentially lifelong difficulty in reading fluency remains a key indicator of dyslexia. However, despite this consensus, converging evidence from multiple risk factors (including weaknesses in phonological skills (including RAN), decoding, and oral language, alongside an assessment of family risk, Wagner & Lonigan, 2023) is likely to afford greater reliability and confidence in assigning the label ‘dyslexia’ than either the exclusive use of reading fluency and spelling test score cut‐off criteria or single criterion models such as IQ‐attainment discrepancy or single deficit theories. Evidence for identification will be strengthened further when there is poor response to instruction and intervention, and in such cases, the presence of co‐occurring conditions needs consideration. This assessment approach, which examines and weighs up accumulating risks, therefore also offers a rationale for modes of intervention, depending on the impact of those accrued factors and the stage of literacy acquisition at which they are experienced. Similar arguments have been made for screening for dyslexia by Vaughn et al. (2024) who emphasize the need to assess response to instruction before embarking on diagnostic assessment. Their proposal fits well with the consensus of the Delphi panel's developmental perspective and depends upon the age and the stage of the person with suspected dyslexia. Thus, for young children, the emphasis should be on assigning risk/no risk within a multitiered system of support embodying universal screening; following instruction, there would follow progress monitoring and further assessment when response to intervention is poor. Early predictors include measures of letter knowledge, phoneme awareness, and RAN, and later in development, direct assessment of reading, language, and comprehension processes (see Holden et al. (2025) for further discussion).

One issue that remains controversial is the role of intellectual abilities in the characterization of dyslexia (and relatedly, whether the term can be usefully applied to a person with intellectual disability). This issue has been addressed differently by previous definitions and is an issue that Catts et al. (2024) did not reach agreement upon. In the current study, we gathered a sub‐panel for discussion on these issues after the survey to ensure we had considered the issues thoroughly.

In the Rose definition, it is stated that dyslexia “occurs across the range of intellectual abilities”, but the statement does not necessitate that intellectual abilities have no bearing on diagnosis, treatment, or support for individuals. We prefer to consider intellectual abilities as part of the multi‐factorial context described above. Hence, a discrepancy between intellectual abilities and reading and spelling attainments (unexpectedness) is a potential indicator of dyslexia but should not be considered necessary or sufficient for diagnosis. Similarly, views differ as to whether it is useful to diagnose ‘dyslexia’ in an individual with intellectual disability. ICD‐11 suggests the term could be used if reading levels are below expectation given the general cognitive problems that are experienced by such individuals. However, few standardized measures have low enough floors to capture variability in reading abilities for individuals with ID. Further, we suggest that the diagnosis of dyslexia in the presence of intellectual disability might result in too narrow an approach to intervention.

A strength of the Delphi study was direct collaboration between individuals with different perspectives on dyslexia. This approach highlighted the different views about dyslexia in research, practice, and personal experience of the condition. An issue that has arisen is the distinction between a definition of dyslexia and the process of diagnosis, assessment, and intervention. While closely related, these issues are separable and important to clinical practice (see Holden et al., 2025). Researchers (current authors included) have sometimes assumed that to select individuals with dyslexia to participate in research, a brief measure of reading is an adequate proxy. However, we maintain that not all poor readers are dyslexic. It remains important to exclude other potential factors that may cause reading difficulties if we wish to inform practice in the field.

A limitation of the study is that it did not address the psychosocial impact of dyslexia and hence its impact on motivation, self‐esteem, and emotional well‐being (see Donolato, Cardillo, Mammarella, & Melby‐Lervåg, 2022 for a meta‐analysis). An important avenue for further research is in understanding the extent to which spelling difficulties align with or are separable from reading difficulties in different ages and contexts. While there was some support for difficulties in spelling accuracy and fluency being important factors in dyslexia, there is currently little consensus on how spelling fluency should be assessed (Côté, Breadmore, & Deacon, 2023), which makes it difficult to include spelling fluency in the definition of dyslexia. Future research needs to consider further the complex, multifactorial nature of dyslexia, how it changes over time and contexts, and the understudied relationship of spelling to reading problems.

## Ethical considerations

Ethical clearance for the study was obtained from the Coventry University Ethics Committee, where the first author was based during data collection. All participants gave informed consent for their participation in the survey. Data collection and management were in line with GDPR requirements.

**Key points**

- Dyslexia is a neurodevelopmental disorder characterized by a reading disability which is highly heritable and often unexpected given other cognitive abilities.
- We propose a definition of dyslexia that emphasizes its multifactorial basis and its persistence through development despite changes in manifestation.
- Key features of dyslexia across all languages are problems acquiring reading and writing fluency.
- Major risk factors are a family history of dyslexia, poor language, phonological difficulties, and persistence of difficulties despite intervention.
- We recommend that assessments for dyslexia should take a developmental perspective and be conducted within a framework that incorporates the assessment of significant risk factors.
- All individuals with literacy difficulties require support and intervention, regardless of cause.

## Data Availability

The anonymized data and R code for reports are freely available via the open science framework: https://osf.io/vhxgf/.

## References

[jcpp14123-bib-0001] American Psychiatric Association . (2013). Diagnostic and statistical manual of mental disorders: DSM‐5 (Vol. 5, No. 5). Washington, DC: Author.

[jcpp14123-bib-0002] Araújo, S. , & Faísca, L. (2019). A meta‐analytic review of naming‐speed deficits in developmental dyslexia. Scientific Studies of Reading, 23, 349–368.

[jcpp14123-bib-0003] Barrett, D. , & Heale, R. (2020). What are Delphi studies? Evidence‐Based Nursing, 23, 68–69. 32430290 10.1136/ebnurs-2020-103303

[jcpp14123-bib-0004] Bishop, D.V. , Snowling, M.J. , Thompson, P.A. , Greenhalgh, T. , & Catalise Consortium . (2016). CATALISE: A multinational and multidisciplinary Delphi consensus study. Identifying language impairments in children. PLoS One, 11, e0158753. 27392128 10.1371/journal.pone.0158753 PMC4938414

[jcpp14123-bib-0005] Bishop, D.V. , Snowling, M.J. , Thompson, P.A. , Greenhalgh, T. , Catalise‐2 Consortium , Adams, C. , & House, A. (2017). Phase 2 of CATALISE: A multinational and multidisciplinary Delphi consensus study of problems with language development: Terminology. Journal of Child Psychology and Psychiatry, 58, 1068–1080. 28369935 10.1111/jcpp.12721 PMC5638113

[jcpp14123-bib-0006] Bradshaw, A.R. , Woodhead, Z.V. , Thompson, P.A. , & Bishop, D.V. (2021). Profile of language abilities in a sample of adults with developmental disorders. Dyslexia, 27, 3–28. 33200857 10.1002/dys.1672 PMC7894539

[jcpp14123-bib-0007] Burt, J.S. (2006). What is orthographic processing skill and how does it relate to word identification in reading? Journal of Research in Reading, 29, 400–417.

[jcpp14123-bib-0008] Callens, M. , Tops, W. , & Brysbaert, M. (2012). Cognitive profile of students who enter higher education with an indication of dyslexia. PLoS One, 7, e38081. 22719864 10.1371/journal.pone.0038081 PMC3374824

[jcpp14123-bib-0009] Caravolas, M. , Lervåg, A. , Defior, S. , Seidlová Málková, G. , & Hulme, C. (2013). Different patterns, but equivalent predictors, of growth in reading in consistent and inconsistent orthographies. Psychological Science, 24, 1398–1407. 23744876 10.1177/0956797612473122

[jcpp14123-bib-0010] Carroll, J.M. , Maughan, B. , Goodman, R. , & Meltzer, H. (2005). Literacy difficulties and psychiatric disorders: Evidence for comorbidity. Journal of Child Psychology and Psychiatry, 46, 524–532. 15845132 10.1111/j.1469-7610.2004.00366.x

[jcpp14123-bib-0011] Carroll, J.M. , Mundy, I.R. , & Cunningham, A.J. (2014). The roles of family history of dyslexia, language, speech production and phonological processing in predicting literacy progress. Developmental Science, 17, 727–742. 24581037 10.1111/desc.12153

[jcpp14123-bib-0012] Carroll, J.M. , Solity, J. , & Shapiro, L.R. (2016). Predicting dyslexia using prereading skills: The role of sensorimotor and cognitive abilities. Journal of Child Psychology and Psychiatry, 57, 750–758. 26662375 10.1111/jcpp.12488 PMC4991277

[jcpp14123-bib-0015] Catts, H.W. , Terry, N.P. , Lonigan, C.J. , Compton, D.L. , Wagner, R.K. , Steacy, L.M. , & Petscher, Y. (2024). Revisiting the definition of dyslexia. Annals of Dyslexia, 74, 1–21. 38194056 10.1007/s11881-023-00295-3 PMC12063701

[jcpp14123-bib-0016] Chamberlain, R. , Brunswick, N. , Siev, J. , & McManus, I.C. (2018). Meta‐analytic findings reveal lower means but higher variances in visuospatial ability in dyslexia. British Journal of Psychology, 109, 897–916. 29938776 10.1111/bjop.12321

[jcpp14123-bib-0017] Côté, E. , Breadmore, H.L. , & Deacon, S.H. (2023). It's about the process, not perfection: What spelling fluency tells us about spelling. Routledge International Handbook of visual‐motor skills, handwriting and spelling (pp. 49–62). London: Routledge.

[jcpp14123-bib-0018] Deacon, S.H. , Benere, J. , & Castles, A. (2012). Chicken or egg? Untangling the relationship between orthographic processing skill and reading accuracy. Cognition, 122, 110–117. 22030120 10.1016/j.cognition.2011.09.003

[jcpp14123-bib-0019] Donolato, E. , Cardillo, R. , Mammarella, I.C. , & Melby‐Lervåg, M. (2022). Research Review: Language and specific learning disorders in children and their co‐occurrence with internalizing and externalizing problems: A systematic review and meta‐analysis. Journal of Child Psychology and Psychiatry, 63, 507–518. 34747025 10.1111/jcpp.13536

[jcpp14123-bib-0020] Dyck, M.J. , Hay, D. , Anderson, M. , Smith, L.M. , Piek, J. , & Hallmayer, J. (2004). Is the discrepancy criterion for defining developmental disorders valid? Journal of Child Psychology and Psychiatry, 45, 979–995. 15225340 10.1111/j.1469-7610.2004.t01-1-00290.x

[jcpp14123-bib-0021] Elliott, J.G. (2020). It's time to be scientific about dyslexia. Reading Research Quarterly, 55, S61–S75.

[jcpp14123-bib-0022] Elliott, J.G. , & Grigorenko, E.L. (2014). The dyslexia debate. Cambridge: Cambridge University Press.

[jcpp14123-bib-0023] Ellis, A.W. (1985). The cognitive neuropsychology of developmental (and acquired) dyslexia: A critical survey. Cognitive Neuropsychology, 2, 169–205.

[jcpp14123-bib-0024] Erbeli, F. , Peng, P. , & Rice, M. (2022). No evidence of creative benefit accompanying dyslexia: A meta‐analysis. Journal of Learning Disabilities, 55, 242–253. 33899570 10.1177/00222194211010350

[jcpp14123-bib-0025] Ferrer, E. , Shaywitz, B.A. , Holahan, J.M. , & Shaywitz, S.E. (2023). Early reading at first grade predicts adult reading at age 42 in typical and dyslexic readers. npj Science of Learning, 8, 51. 38016979 10.1038/s41539-023-00205-7 PMC10684638

[jcpp14123-bib-0026] Georgiou, G.K. , Martinez, D. , Vieira, A.P.A. , & Guo, K. (2021). Is orthographic knowledge a strength or a weakness in individuals with dyslexia? Evidence from a meta‐analysis. Annals of Dyslexia, 71, 5–27. 33712993 10.1007/s11881-021-00220-6

[jcpp14123-bib-0027] Griffiths, P.G. , Taylor, R.H. , Henderson, L.M. , & Barrett, B.T. (2016). The effect of coloured overlays and lenses on reading: A systematic review of the literature. Ophthalmic and Physiological Optics, 36, 519–544. 27580753 10.1111/opo.12316

[jcpp14123-bib-0028] Hinshelwood, J. (1896). A case of dyslexia: A peculiar form of word‐blindness. 1. The Lancet, 148, 1451–1454.

[jcpp14123-bib-0029] Holden, C. , Kirby, P. , Snowling, M.J. , Thompson, P. , Carroll, J.M., & the Dyslexia Delphi Panel. (2025). Towards a consensus for dyslexia practice: Findings of a Delphi study on assessment and identification. Dyslexia, forthcoming. 10.1002/dys.1800 PMC11852196 39996532

[jcpp14123-bib-0030] Hudson, R.F. , Lane, H.B. , Pullen, P.C. , & Torgesen, J.K. (2009). The complex nature of reading fluency: A multidimensional view. Reading and Writing Quarterly, 25, 4–32.

[jcpp14123-bib-0031] IDA . (2002). https://dyslexiaida.org/definition‐of‐dyslexia/

[jcpp14123-bib-0033] Kirby, P. , & Snowling, M.J. (2022). Dyslexia: A history. Montreal: MQUP. 36730539

[jcpp14123-bib-0034] Kormos, J. , & Smith, A.M. (2023). Teaching languages to students with specific learning differences (18). Bristol, UK: Multilingual Matters.

[jcpp14123-bib-0035] Kriss, I. , & Evans, B.J. (2005). The relationship between dyslexia and Meares‐Irlen Syndrome. Journal of Research in Reading, 28, 350–364.

[jcpp14123-bib-0036] Kussmaul, A. (1877). Diseases of the nervous system and disturbances of speech. In H. von Ziemssen (Ed.), Cylopedia of the practice of medicine (pp. 770–778). New York: William Wood.

[jcpp14123-bib-0037] Landerl, K. , & Moll, K. (2010). Comorbidity of learning disorders: Prevalence and familial transmission. Journal of Child Psychology and Psychiatry, 51, 287–294. 19788550 10.1111/j.1469-7610.2009.02164.x

[jcpp14123-bib-0038] Lervåg, A. , & Hulme, C. (2009). Rapid automatized naming (RAN) taps a mechanism that places constraints on the development of early reading fluency. Psychological Science, 20, 1040–1048. 19619178 10.1111/j.1467-9280.2009.02405.x

[jcpp14123-bib-0039] Lyon, G.R. , Shaywitz, S.E. , & Shaywitz, B.A. (2003). A definition of dyslexia. Annals of Dyslexia, 53, 1–14.

[jcpp14123-bib-0040] Lyytinen, H. , Guttorm, T.K. , Huttunen, T. , Hämäläinen, J. , Leppänen, P.H. , & Vesterinen, M. (2005). Psychophysiology of developmental dyslexia: A review of findings including studies of children at risk for dyslexia. Journal of Neurolinguistics, 18, 167–195.

[jcpp14123-bib-0041] Majeed, N.M. , Hartanto, A. , & Tan, J.J. (2021). Developmental dyslexia and creativity: A meta‐analysis. Dyslexia, 27, 187–203. 33586314 10.1002/dys.1677

[jcpp14123-bib-0042] Maughan, B. , Messer, J. , Collishaw, S. , Pickles, A. , Snowling, M. , Yule, W. , & Rutter, M. (2009). Persistence of literacy problems: Spelling in adolescence and at mid‐life. Journal of Child Psychology and Psychiatry, 50, 893–901. 19490310 10.1111/j.1469-7610.2009.02079.x

[jcpp14123-bib-0043] McBride, C. , Pan, D.J. , & Mohseni, F. (2022). Reading and writing words: A cross‐linguistic perspective. Scientific Studies of Reading, 26, 125–138.

[jcpp14123-bib-0044] McGrath, L.M. , Peterson, R.L. , & Pennington, B.F. (2020). The multiple deficit model: Progress, problems, and prospects. Scientific Studies of Reading, 24, 7–13. 32440085 10.1080/10888438.2019.1706180 PMC7241589

[jcpp14123-bib-0045] Melby‐Lervåg, M. , Lyster, S.A.H. , & Hulme, C. (2012). Phonological skills and their role in learning to read: A meta‐analytic review. Psychological Bulletin, 138, 322–352. 22250824 10.1037/a0026744

[jcpp14123-bib-0046] Moll, K. , Snowling, M.J. , & Hulme, C. (2020). Introduction to the special issue “comorbidities between reading disorders and other developmental disorders”. Scientific Studies of Reading, 24, 1–6.

[jcpp14123-bib-0047] Nag, S. (2022). Reading the Akshara writing system. In M.H. Snowling , C. Hulme , & K. Nation (Eds.), The science of reading: A handbook (pp. 372–389). Oxford, UK: Wiley Blackwell.

[jcpp14123-bib-0048] Nation, K. , & Snowling, M.J. (1998). Individual differences in contextual facilitation: Evidence from dyslexia and poor reading comprehension. Child Development, 69, 996–1011. 9768483

[jcpp14123-bib-0049] Orton, S.T. (1925). “Word blindness” in schoolchildren. Archives of Neurology and Psychiatry, 14, 581–615.

[jcpp14123-bib-0050] Packheiser, J. , Papadatou‐Pastou, M. , Koufaki, A. , Paracchini, S. , Stein, C.C. , Schmitz, J. , & Ocklenburg, S. (2023). Elevated levels of mixed‐hand preference in dyslexia: Meta‐analyses of 68 studies. Neuroscience & Biobehavioral Reviews, 154, 105420. 37783301 10.1016/j.neubiorev.2023.105420

[jcpp14123-bib-0051] Pennington, B.F. (2006). From single to multiple deficit models of developmental disorders. Cognition, 101, 385–413. 16844106 10.1016/j.cognition.2006.04.008

[jcpp14123-bib-0052] Poulsen, M. , Juul, H. , & Elbro, C. (2015). Multiple mediation analysis of the relationship between rapid naming and reading. Journal of Research in Reading, 38, 124–140.

[jcpp14123-bib-0053] Pringle Morgan, W. (1896). A case of congenital word blindness. Lancet, 2, 1378. 10.1136/bmj.2.1871.1378 PMC2510936 20756570

[jcpp14123-bib-0054] Protopapas, A. , Altani, A. , & Georgiou, G.K. (2013). Development of serial processing in reading and rapid naming. Journal of Experimental Child Psychology, 116, 914–929. 24077466 10.1016/j.jecp.2013.08.004

[jcpp14123-bib-0055] Pugh, K. , & Verhoeven, L. (2018). Introduction to this special issue: Dyslexia across languages and writing systems. Scientific Studies of Reading, 22, 1–6.

[jcpp14123-bib-0056] R Core Team . (2023). R: A language and environment for statistical computing. Vienna, Austria: R Foundation for Statistical Computing. https://www.R‐project.org/

[jcpp14123-bib-0057] Ramus, F. , Rosen, S. , Dakin, S.C. , Day, B.L. , Castellote, J.M. , White, S. , & Frith, U. (2003). Theories of developmental dyslexia: Insights from a multiple case study of dyslexic adults. Brain, 126, 841–865. 12615643 10.1093/brain/awg076

[jcpp14123-bib-0058] Rooke, M. (2017). Dyslexia is my superpower (most of the time). London, UK: Jessica Kingsley Publishers.

[jcpp14123-bib-0059] Rose, J. (2009). Identifying and teaching children with dyslexia and other literacy difficulties. Available from: https://www.thedyslexia‐spldtrust.org.uk/media/downloads/inline/the‐rose‐report.1294933674.pdf

[jcpp14123-bib-0060] SASC . (2022). Specific Learning Difficulties (SpLD) Assessment Standards Committee (SASC) Consultation Paper on the identification of and effective intervention for literacy difficulties in children and adults. Implications for the assessment of dyslexia. Available from: https://www.sasc.org.uk/media/a3zkvkqa/holden‐survey_presentation‐2022‐06‐08.pdf

[jcpp14123-bib-0061] Scarborough, H.S. (1990). Very early language deficits in dyslexic children. Child Development, 61, 1728–1743. 2083495

[jcpp14123-bib-0063] Share, D.L. (2008). On the Anglocentricities of current reading research and practice: The perils of overreliance on an “outlier” orthography. Psychological Bulletin, 134, 584–615. 18605821 10.1037/0033-2909.134.4.584

[jcpp14123-bib-0064] Shaywitz, S.E. , Fletcher, J.M. , Holahan, J.M. , Shneider, A.E. , Marchione, K.E. , Stuebing, K.K. , & Shaywitz, B.A. (1999). Persistence of dyslexia: The Connecticut longitudinal study at adolescence. Pediatrics, 104, 1351–1359. 10585988 10.1542/peds.104.6.1351

[jcpp14123-bib-0066] Snowling, M.J. , Gallagher, A. , & Frith, U. (2003). Family risk of dyslexia is continuous: Individual differences in the precursors of reading skill. Child Development, 74, 358–373. 12705560 10.1111/1467-8624.7402003

[jcpp14123-bib-0068] Snowling, M.J. , Hulme, C. , & Nation, K. (2020). Defining and understanding dyslexia: Past, present and future. Oxford Review of Education, 46(4), 501–513. 32939103 10.1080/03054985.2020.1765756 PMC7455053

[jcpp14123-bib-0069] Snowling, M.J. , Hulme, C. , & Nation, K. (Eds.). (2022). The science of reading: A handbook. Oxford, UK: John Wiley & Sons.

[jcpp14123-bib-0070] Snowling, M.J. , Muter, V. , & Carroll, J. (2007). Children at family risk of dyslexia: A follow‐up in early adolescence. Journal of Child Psychology and Psychiatry, 48, 609–618. 17537077 10.1111/j.1469-7610.2006.01725.x

[jcpp14123-bib-0071] Snowling, M.J. , Nash, H.M. , Gooch, D.C. , Hayiou‐Thomas, M.E. , Hulme, C. , & Wellcome Language and Reading Project Team . (2019). Developmental outcomes for children at high risk of dyslexia and children with developmental language disorder. Child Development, 90, e548–e564. 30676649 10.1111/cdev.13216 PMC6767399

[jcpp14123-bib-0072] Spencer, M. , Wagner, R.K. , Schatschneider, C. , Quinn, J. , Lopez, D. , & Petscher, Y. (2014). Incorporating RTI in a hybrid model of reading disability. Learning Disability Quarterly, 37, 161–171. 25422531 10.1177/0731948714530967 PMC4240020

[jcpp14123-bib-0073] Stanovich, K.E. , & Siegel, L.S. (1994). Phenotypic performance profile of children with reading disabilities: A regression‐based test of the phonological‐core variable‐difference model. Journal of Educational Psychology, 86, 24–53.

[jcpp14123-bib-0074] Thompson, A. (2021). The illusion of inclusion: how parents of children with dyslexia perceive, understand and enact inclusion . PhD thesis, Coventry University.

[jcpp14123-bib-0075] Thompson, P.A. , Hulme, C. , Nash, H.M. , Gooch, D. , Hayiou‐Thomas, E. , & Snowling, M.J. (2015). Developmental dyslexia: Predicting individual risk. Journal of Child Psychology and Psychiatry, 56, 976–987. 25832320 10.1111/jcpp.12412 PMC4672694

[jcpp14123-bib-0076] Torppa, M. , Eklund, K. , van Bergen, E. , & Lyytinen, H. (2015). Late‐emerging and resolving dyslexia: A follow‐up study from age 3 to 14. Journal of Abnormal Child Psychology, 43, 1389–1401. 25772426 10.1007/s10802-015-0003-1

[jcpp14123-bib-0077] Vaughn, S. , Miciak, J. , Clemens, N. , & Fletcher, J. M. (2024). The critical role of instructional response in defining and identifying students with dyslexia: a case for updating existing definitions. Annals of Dyslexia, 74, 325–336. 10.1007/s11881-024-00303-0. 38526758

[jcpp14123-bib-0083] von Hagen, A. , Kohnen, S. , & Stadie, N. (2021). Foreign language attainment of children/adolescents with poor literacy skills: A systematic review and meta‐analysis. Educational Psychology Review, 33, 459–488.

[jcpp14123-bib-0078] Wagner, R.K. , & Lonigan, C.J. (2023). Early identification of children with dyslexia: Variables differentially predict poor reading versus unexpected poor reading. Reading Research Quarterly, 58, 188–202. 37448987 10.1002/rrq.480 PMC10338016

[jcpp14123-bib-0079] Wagner, R.K. , Torgesen, J.K. , Laughon, P. , Simmons, K. , & Rashotte, C.A. (1993). Development of young readers' phonological processing abilities. Journal of Educational Psychology, 85, 83–103.

[jcpp14123-bib-0080] Wagner, R.K. , Zirps, F.A. , Edwards, A.A. , Wood, S.G. , Joyner, R.E. , Becker, B.J. , … & Beal, B. (2020). The prevalence of dyslexia: A new approach to its estimation. Journal of Learning Disabilities, 53, 354–365. 32452713 10.1177/0022219420920377 PMC8183124

[jcpp14123-bib-0081] World Health Organization . (2018). International classification of diseases for mortality and morbidity statistics (11th Revision). Available from: https://icd.who.int/browse11/l‐m/en

[jcpp14123-bib-0082] Yeatman, J.D. (2022). The neurobiology of literacy. In The science of reading: A handbook (pp. 533–555). Hoboken, NJ: Blackwell.

